# Lyotropic Liquid Crystal Phases from Anisotropic Nanomaterials

**DOI:** 10.3390/nano7100305

**Published:** 2017-10-01

**Authors:** Ingo Dierking, Shakhawan Al-Zangana

**Affiliations:** 1 School of Physics and Astronomy, University of Manchester, Oxford Road, Manchester M13 9PL, UK; 2 College of Education, University of Garmian, Kalar 46021, Iraq; shakhawan.al-zangana@hotmail.com

**Keywords:** liquid crystal, lyotropic, inorganic nanoparticle, clay, tobacco mosaic virus (TMV), Deoxyribonucleic acid (DNA), cellulose nanocrystal, nanotube, nanowire, nanorod, graphene, graphene oxide

## Abstract

Liquid crystals are an integral part of a mature display technology, also establishing themselves in other applications, such as spatial light modulators, telecommunication technology, photonics, or sensors, just to name a few of the non-display applications. In recent years, there has been an increasing trend to add various nanomaterials to liquid crystals, which is motivated by several aspects of materials development. (i) addition of nanomaterials can change and thus tune the properties of the liquid crystal; (ii) novel functionalities can be added to the liquid crystal; and (iii) the self-organization of the liquid crystalline state can be exploited to template ordered structures or to transfer order onto dispersed nanomaterials. Much of the research effort has been concentrated on thermotropic systems, which change order as a function of temperature. Here we review the other side of the medal, the formation and properties of ordered, anisotropic fluid phases, liquid crystals, by addition of shape-anisotropic nanomaterials to isotropic liquids. Several classes of materials will be discussed, inorganic and mineral liquid crystals, viruses, nanotubes and nanorods, as well as graphene oxide.

## 1. Introduction

Liquid crystals (LC) are a state of matter which is thermodynamically located between the isotropic liquid and the crystalline phase [1,2]. They exhibit flow properties like a liquid and at the same time partially retain the order of a crystal. For this reason, they possess anisotropic physical properties such as their refractive index, dielectric constant, elastic behaviour, or viscosity, just to name a few. But while being partially ordered, LCs also exhibit flow properties like a liquid; they are thus anisotropic fluids. The liquid crystalline state can be brought about via two fundamentally different ways, leading to the two basic classes of LC, thermotropic phases and lyotropic phases. The former is achieved by varying an intensive variable of state, such as temperature or pressure, while the latter is formed through a variation of the concentration of a dopant in an isotropic solvent, often water.

### 1.1. Thermotropic Liquid Crystals

**Figure 1:**
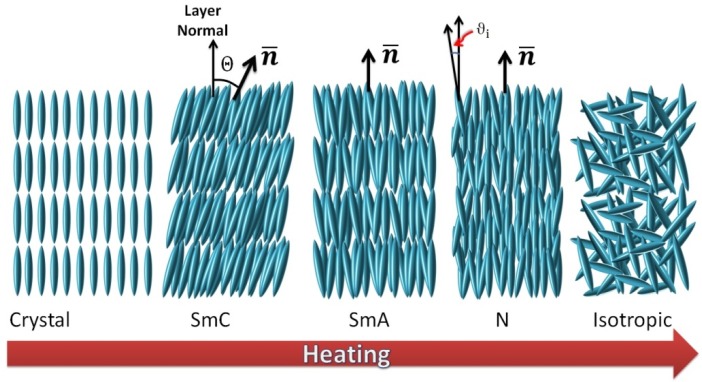
Schematic illustration of different liquid crystal (LC) phases observed on heating from the crystalline state. At first, positional ordering is partially maintained in the smectic phases, SmC and SmA, together with orientational order of the long molecular axis of often rod-shaped molecules. On further heating, positional order is lost at the transition to the nematic phase, which solely exhibits orientational order. Eventually, at the clearing point, all liquid crystalline order is lost and the isotropic liquid is reached. For simplicity, only rod-like molecules are depicted in the figure, but other molecular shapes exist as well, such as disc-like or bent-core materials, which exhibit liquid crystalline behavior.

Thermotropic LCs [3,4,5] are the ones which are widely known due to their applicational impact in flat screen televisions, laptop and tablet displays, or mobile phones [6]. All these applications rely on the fact that LCs exhibit elastic behaviour and can be addressed via electric or magnetic fields, which changes the orientation of the optic axis, and thus the birefringence. Thermotropic LCs are further distinguished by their degree of order, showing further phase transitions within the temperature regime of the liquid crystalline state. The phase generally observed below the isotropic liquid is called nematic, N, and exhibits solely orientational order of the long axis of rod-like molecules, while disk-like molecules, so called discotic, can show nematic phases as well. The spatial and temporal average of this long axis is called the director, ***n***. At lower temperatures smectic phases are also observed, which, in addition to orientational order, also exhibit one- or two-dimensional positional order of the molecules centres of mass. Depending on the degree and nature of order, a whole range of different smectic phases can be distinguished, with the simplest being the smectic A phase with one-dimensional positional order and the director in direction of the smectic layer normal. If the director, which at the same time is the optic axis of the system, is inclined to the layer normal, one speaks of the smectic C phase (see Figure 1).

**Figure 2:**
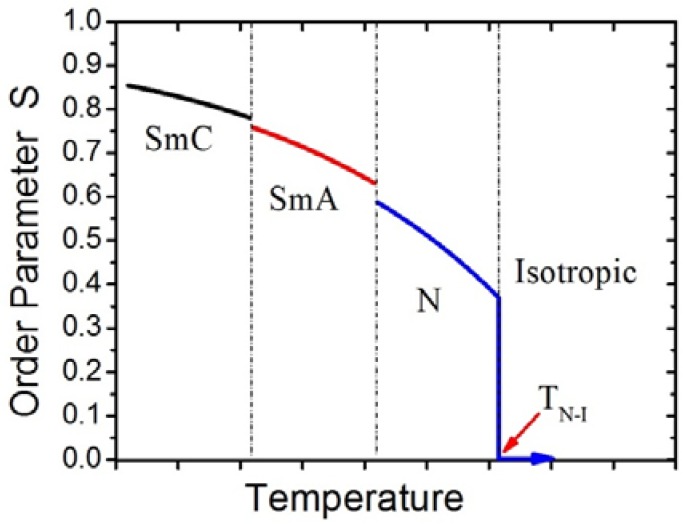
Schematic temperature dependence of the scalar orientational order parameter S. At elevated temperatures in the isotropic phase, it is S = 0. At the clearing temperature T_N-I_, a first order transition into the nematic phase is observed, accompanied by a discontinuous jump of the order parameter, generally to S ≈ 0.45, which then increases with decreasing temperature to value of about S ≈ 0.6–0.7. Further increases in orientational order are observed at the transitions into smectic phases, albeit much smaller than those between the nematic and the isotropic phase.

These phases are called the fluid smectic phases, with hexatic phases and higher ordered phases to follow at even lower temperature [7]. The order thus increases with decreasing temperature, while the symmetry is reduced. An important parameter in the description of LC phases is the orientational order parameter, S_2_, which in its simplest description takes the form:

(1) $$S_{2}=\langle P_{2}\left(cos\vartheta \right)\rangle =\langle \frac{3cos^{2}\vartheta -1}{2}\rangle$$

where *P*_2_ is the second Legendre polynomial and *ϑ* the angle between the long axis of an individual molecule and the director. The order parameter changes as a function of temperature, a dependence which is schematically shown in Figure 2.

### 1.2. Lyotropic Liquid Crystals

**Figure 3:**
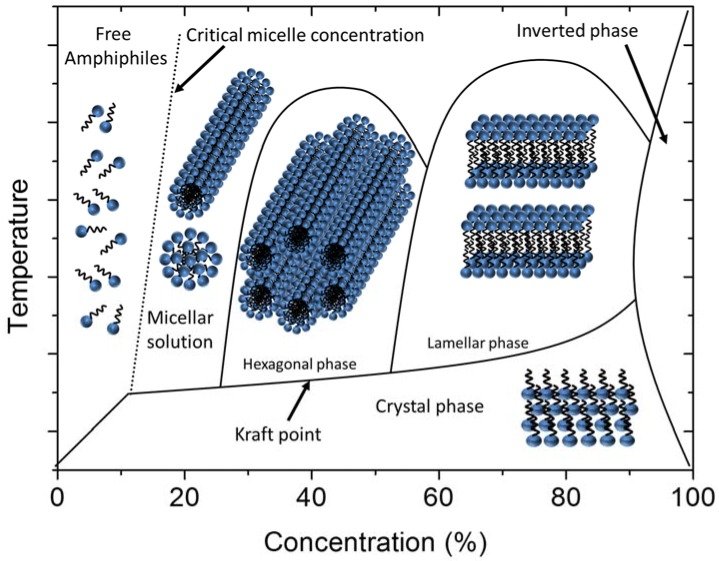
Schematic illustration of the phase diagram of an amphiphilic surfactant in an isotropic solvent, forming lyotropic phases. Crossing the critical micelle concentration, cmc, spherical or cylindrical micelles are formed. At higher surfactant concentrations, these can aggregate to liquid crystalline phases, namely the hexagonal and the lamellar phase, for increasing concentration. Cubic phases, which are not shown in this figure, can occur at different regimes of the phase diagram.

Lyotropic LCs [8,9] on the other hand are observed when changing the concentration of a shape- or property anisotropic dispersant in an isotropic solvent. Often, lyotropic phases are observed as a function of concentration of amphiphilic molecules in water or other solvents, as schematically shown in Figure 3. Below the critical micelle concentration, cmc, the amphiphiles are molecularly dispersed in the solvent, but at larger concentrations form micelles, which can be of the spherical, disk or rod-like type, depending on the molecular shape. At even higher concentrations, these micelles aggregate to ordered structures and can form hexagonal, cubic or lamellar phases, also of the inverse type. The observed phase diagrams can be quite complex, as they depend largely on concentration, but also on temperature.

**Figure 4:**
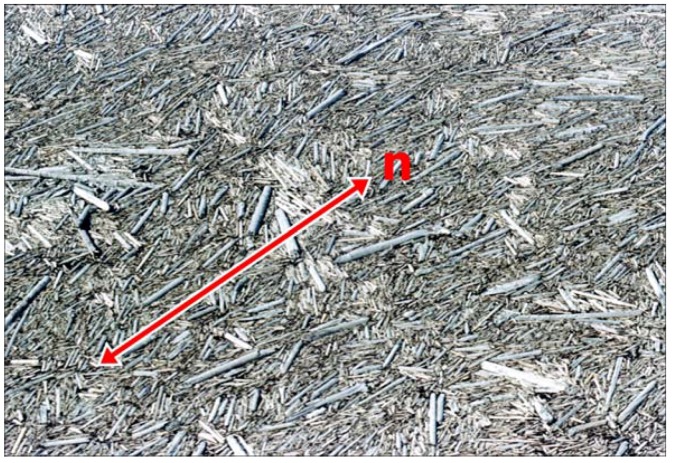
Logs washed into the Spirit Lake after the eruption of the volcano St. Helens in 1980. The photograph was taken from a helicopter by Col. David K. Wendt, USAF Reserves, who was one of the first arriving with a rescue team, one day after the eruption. The logs exhibit the nematic ordering of rigid rods, as proposed by Lars Onsager. (The length of the picture is estimated to approximately 50 m).

Similarly, dispersions of shape-anisotropic nanoparticles, like nanorods or nanoplates, in isotropic solvents can lead to the formation of often nematic lyotropic LC phases. Spontaneous self-organization is observed, such that the dispersed particles order roughly parallel. The structure is very much reminiscent of its two-dimensional analogue of floating trees on the surface of a lake. Figure 4 shows one of the first photographs of Spirit Lake after the eruption of the volcano Mt. St. Helens in 1980, taken by Col. David K. Wendt, USAF Reserves, one day after the explosive eruption. Whole forests were washed into the lake, where the logs collected to form a “nematic” structure with the logs locally pointing approximately in the same direction, minimizing the free volume. This average direction would then be defined as the director, ***n***, in the case of Figure 4 approximately along the diagonal from bottom left to top right.

In general, the colloidal suspensions of geometrically anisotropic particles can be observed to produce a LC phase above a critical concentration. Orientational order arises from particle anisotropy for an associated critical volume fraction V_crit_ depending on the aspect ratio, A_R_ = W/L, of the particle as V_crit_ ≈ 4/A_R_ = 4 L/W, where L is the length and W the width of the nanoparticle. This first theoretical description was reported by Onsager [10]. His theory is based on the fact that when the concentration of particles reaches a certain level, the freedom of the particles is constrained and as a result, the entropy decreases due to overlapping excluded volume of the particles. To compensate for the entropy decrease, the particles start to acquire orientational order. Thus, above a critical concentration orientation order is achieved and a nematic LC phase is formed. First experimental reports go back even further when liquid crystalline behaviour was described for tobacco and tomato mosaic virus (TMV) [11] and vanadium pentoxide (V_2_O_5_) [12] in the early 20th century. Decades later Mourchid et al., studied colloidal plate-like charged particles and clay particles to report liquid crystalline behaviour [13]. In this review, we will try to summarize some of the most interesting experimental systems and properties of liquid crystalline behaviour from shape-anisotropic nanoparticles, such as inorganic and mineral materials, clays, biological nanoparticles, such as TMV, DNA and cellulose nanocrystals, nanotubes and nanorods, as well as nanoplates and two-dimensional materials, such as graphene, graphene oxide, and reduced graphene oxide.

### 1.3. Isotropic to Nematic Transition: Maier-Saupe vs. Onsager

Thermotropic LCs are generally described by the Maier-Saupe theory [14,15,16] , which is based exclusively on attractive interactions of the van der Waals type, thus induced dipole-induced dipole interactions. This works very well, because the rigid, polarizable cores of respective mesogens are mainly of the aromatic type, and steric repulsion can largely be ignored. Through a self-consistent field equation, which has to be solved numerically, the Maier-Saupe theory predicts the temperature dependence of the (scalar) order parameter S_2_. At a certain temperature a discontinuous, first order transition is observed, at which the order parameter takes a value of S_2_ = 0.43, continuously increasing with decreasing temperature, to values of the order of S_2_ = 0.7–0.8 for rather low temperatures. The Maier-Saupe approach does not yield a satisfactory description of lyotropic LCs though, especially those based on colloidal particles as they are discussed in this publication. 

**Figure 5:**
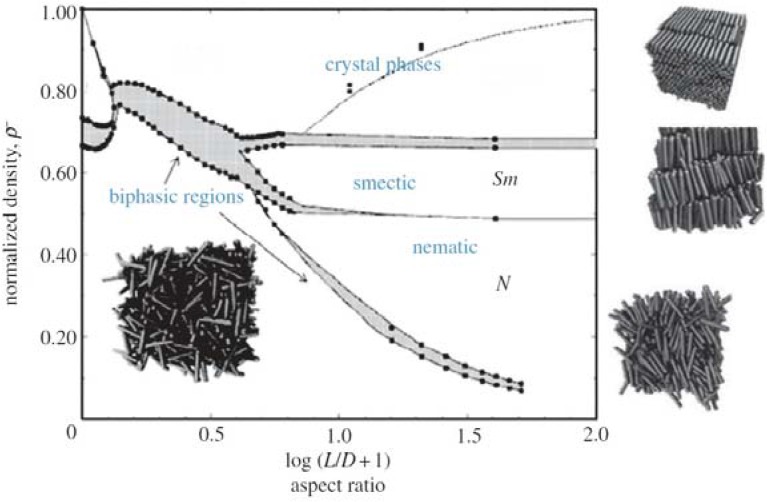
Predicted phase diagram from computer simulations of a rigid rod system, showing the isotropic phase at low aspect ratios and low concentrations, then an Iso + N biphasic region, before a nematic phase is established. Eventually, also smectic and crystalline phases are observed. (Reproduced by permission from ref. [19]).

Already a decade before the work of Maier and Saupe, Onsager [10] formulated a theory which was able to describe the occurrence of a nematic state in colloidal suspensions, and which was largely the opposite of the approach that Maier and Saupe took later on. Onsager’s theory starts from the assumption that no two particles can occupy the same space, all interactions between suspended colloids are in fact repulsive, ignoring any attractive van der Waals forces. Repulsive interactions can be steric, thus based on excluded volume, or they can be of electrostatic nature. Onsager in fact accounted for electrostatic repulsion as approximating it through an increase in particle size, which was later detailed more correctly [17,18]. Further simplifications that were made in the first instance, but relaxed later on (see [18] and references therein), were monodispersity and the use of rigid rod particles of length L and diameter D. Thus, the aspect ratio D/L plays a paramount role in Onsager’s approach. The description is purely based on maximizing the entropy. The phase behaviour of such a colloidal suspension is found by the minimization of the free energy F = U − TS, where the internal energy U = 0 for pure steric repulsion, T is the temperature and S the entropy. There are two competing effects: decreasing the excluded volume increases the free volume and thus the translational entropy of the particles. This on the other hand implies a reduction of the mixing entropy. When the particle concentration is small the mixing entropy term dominates the phase behaviour and the isotropic phase is maintained. According to Onsager this is the case for particle volume fractions of Ψ_iso_ < 3.3 D/L. At this corresponding particle concentration, a first order transition is observed into the two-phase region of coexisting isotropic and nematic phase. This two-phase region terminates into the nematic phase at a particle volume fraction of Ψ_n_ > 4.5 D/L. A complete phase diagram for rigid rod cylinders from computer simulation by Bolhuis and Frenkel [19] is depicted in Figure 5, which includes not only the isotropic and the nematic phase, but also smectic ordering and colloidal crystals.

The Onsager model leads to very high values of the orientational order parameter of S_2_ ≈ 0.8 at the beginning of the transition into the nematic state, to S_2_ ≈ 0.95 at larger nanoparticle concentrations. These values are much larger than those predicted for thermotropic nematic phases by the Maier-Saupe theory, but are indeed observed for lyotropic phases from anisotropic colloidal particles, as we will see below in an example of the TMV. It is believed that the Maier-Saupe model is more applicable to systems that are only slightly compressible, thus only show a small change of density at the transition and smaller orientational order parameters, while the Onsager model is most appropriately applied to dilute suspensions of particles, showing large changes in density and orientational order parameter at the transition to the liquid crystalline state.

## 2. Lyotropic Phases from Nanomaterials

Much effort has been invested into the study of nanoparticles dispersed in LCs. These systems are mainly studied for their possibility to tune the liquid crystalline properties, such as threshold voltage V_th_, response times τ, viscosity η, dielectric anisotropy Δε, refractive index n, or the birefringence Δn, which are of importance for applications, especially in the area of LC displays (LCDs). Nanoparticles employed in such dispersions are often based on inorganic or mineral materials [20,21] such as dielectric and ferroelectric particles like TiO_2_ [22] and Sn_2_P_2_S_6_ [23] or BaTiO_3_ [24,25], gold nanoparticles [26] or carbon based nanotubes [27,28] and graphene oxide [29]. However, also other nanotubes, like ZnO [30] or semiconducting CdSe [31] have been employed to add additional functionality to the LC matrix. Not surprisingly, many of the shape-anisotropic dopants can also form LC phases by themselves, through dispersion in isotropic solvents. The shape-anisotropy may be provided through the nanoparticles being rod- or tube-like, disk-like or existing as sheet-like materials. These are then representing lyotropic LC phases, similar to the ones formed by amphiphilic molecules dispersed at relatively large concentrations above the cmc in a solvent, often water.

Having shortly outlined above the main predictions and descriptions expected from the theory first devised by Lars Onsager in 1949 [10], we can step back in time for a few decades to discuss the first experimentally observed liquid crystalline systems of this kind.

### 2.1. Inorganic and Mineral Liquid Crystals

LC phases from anisotropic, inorganic or mineral crystallites in a solvent, often water, represent a sol with particles of colloidal size, i.e., particles with at least one dimension smaller than approximately one micrometer. The first studies of such materials go back to 1902 when sols of FeOOH were shown to become birefringent under the application of a magnetic field, today known as the Majorana effect [32]. A few years later, in 1915–1916, Freundlich [12,33] demonstrated on colloidal dispersions of vanadium pentoxide, V_2_O_5_, the occurrence of birefringence induced by flow alignment of anisotropic crystallites, as well as by applied electric field, and concluded that the mechanism of both effects was the same; application of a force to align the long axis of the anisotropic particles leads to an induced birefringence. Removal of the external stimuli causes a thermal relaxation back to an isotropic distribution of particles without any birefringence.

**Figure 6:**
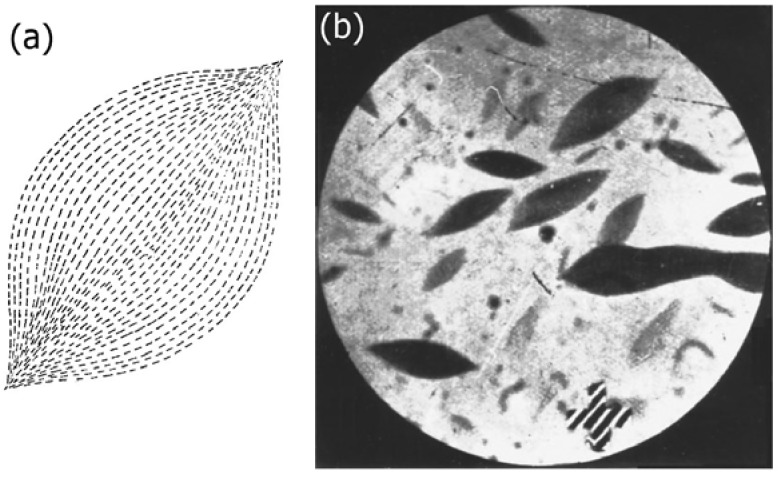
(**a**) schematic illustration of the director/particle field within a nematic tactoid; and (**b**) microscopic photograph of the same for the inorganic LC vanadium pentoxide, V_2_O_5_. (Reproduced by permission from ref. [34]).

It has been reported repeatedly that freshly prepared V_2_O_5_ sols show no birefringence at early times, while only later on, after days, sometimes weeks, anisotropic, birefringent regions develop in the form of tactoids [34]. These are domains with orientationally ordered rod-like particles as shown schematically in Figure 6a. Their shape is clearly different from the circular domains of thermotropic nematics forming at the isotropic to nematic transition, which exhibits round domains due to the minimization of surface tension. Tactoids, which are shown in microscopic observation in Figure 6b for V_2_O_5_, on the other hand exhibit two tips which may be rounded off if flow or electric and magnetic field application is involved. The growth of tactoids has also been reported for other inorganic sols [20] like H_2_WO_4_ and FeOOH, but also for other systems, like chromonic LCs [35,36] or TMV [37]. For inorganic or mineral sols, nematic liquid crystalline behaviour with purely orientational order has been observed, but also smectic type phases with additional ordering in layers can be found.

**Figure 7:**
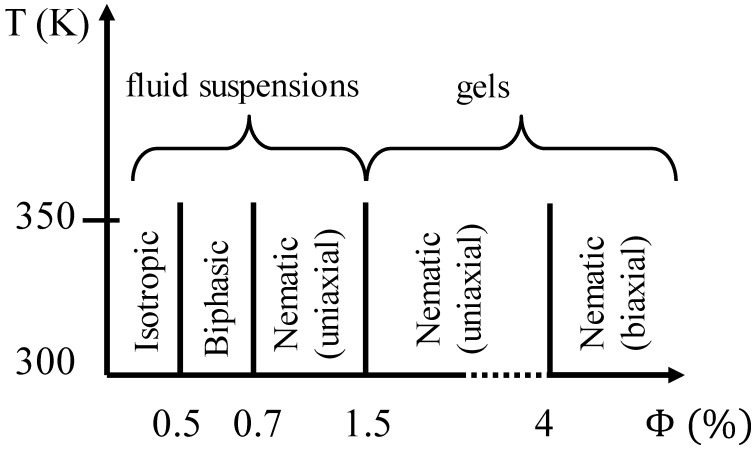
Summary of the phase behaviour of V_2_O_5_ in dependence on the volume fraction of the dispersed inorganic crystallites, as determined by Nuclear Magnetic Resonance, NMR. (Reproduced by permission from ref. [39]).

The classic inorganic LC is vanadium pentoxide V_2_O_5_, which forms a nematic lyotropic phase. The phase formation is strongly dependent on the preparation conditions of V_2_O_5_, which shows small elongated crystallites or fibre like ribbons. This is related to the aging of virgin preparations over time periods of hours or even days, depending on temperature, concentration and electrolyte addition. The aging process, thus the formation process of a nematic phase from the sol, increases in speed for large crystallite concentrations, higher temperatures, and increased electrolyte addition. The particle length then increases at practically constant width of approximately 10 nm from several nanometers to a few micrometers, which is accompanied by a sol-gel transition [38]. Electric field experiments in the nematic phase indicate a negative dielectric anisotropy, Δε < 0. A respective temperature-concentration stability diagram is shown for V_2_O_5_ in Figure 7.

**Figure 8:**
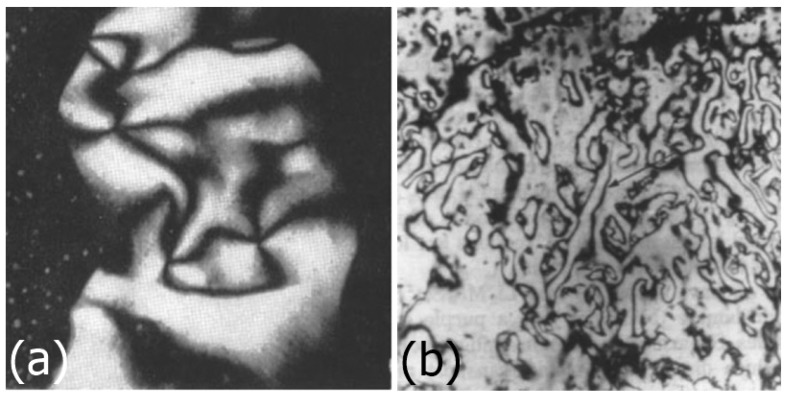
(**a**) Nematic Schlieren texture of AlOOH (reproduced by permission from ref. [40]); and (**b**) nematic thread-like texture of Li_2_Mo_6_Se_6_ (reproduced by permission from ref. [42]), scale unknown.

Similar behaviour as for vanadium pentoxide is observed for aluminium oxyhydroxide, AlOOH, with tactoids of nematic order forming. When these tactoids join, a Schlieren texture with typical disclinations is developed, as shown in Figure 8a [40]. Another nanomaterial to mention is uranyl fluoride UO_2_F_2_. This exhibits a nematic phase in a solution of acetone and heavy water, as demonstrated by nuclear magnetic resonance (NMR) experiments [41].

A general class of inorganic nematic LCs is M_2_Mo_6_X_6_ with the metal M = Li, Na, K from the alkalimetal group 1, and X = Se, Te from the chalcogens group 16. Also here we observe crystallite lengths of a few micrometers and the formation of Schlieren textures or thread-like textures [42], as shown in Figure 8b, clearly identifying nematic behaviour, in this case with N-methylformamide as solvent. The phase separation into a nematic and an isotropic component is observed after several hours to months.

As mentioned above, also the formation of smectic phases can be observed. This has been demonstrated for example for FeOOH by the microscopic observation of step textures indicating smectic layering [43], and for tungstic acid H_2_WO_4_ (WO_3_·H_2_O). A detailed overview about preparation methods and conditions of elongated inorganic particles and their LC structures and phases can be found in a review article by Sonin [20].

### 2.2. Clay Based Liquid Crystals

Clays are obviously a very closely related topic, as they are aluminosilicates, rock-forming minerals. They generally exist in powder form with layered structures made of plates or platelets. This structure is also the reason why they easily swell in the presence of water or other isotropic solvents [44], which are situated between the sheets. Similar to the hard-rod model of Onsager and its later variants, such a model can also be formulated for hard disks, and computer simulations by Veerman and Frenkel indicate a stable nematic and a columnar phase [45].

#### 2.2.1. Bentonite 

Already Langmuir [46] in 1938 observed that a bentonite clay suspension phase separates into an isotropic region and an anisotropic, birefringent region, which in fact turns out to be a gel. Only much later it was realized that at very low concentrations a real LC phase and not a gel could be produced. Bentonite clay is used in a number of diverse applications from food additives to cosmetics, facial masks, and nutritional products, all the way to cat litter and drilling mud. The general application of the liquid crystalline phase may be found in the ordering of inorganic nanosheets for possible future applications in sensors or energy storage. Hybrids with dyes could be used for photosensitization, or in optical materials for plasmonic nanostructures.

#### 2.2.2. Laponite

**Figure 9:**
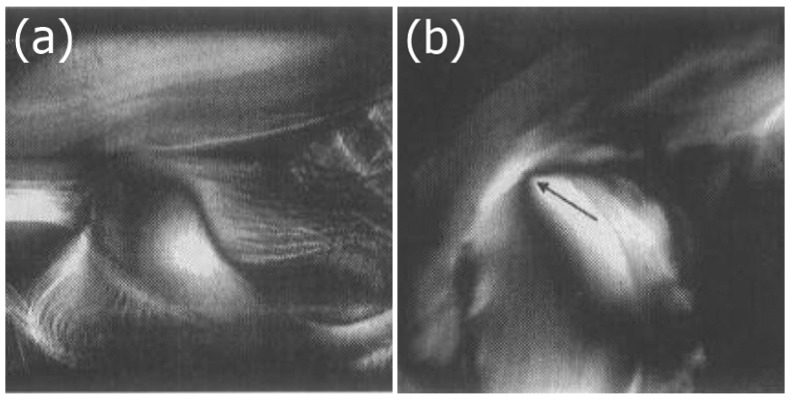
Nematic texture of different clays, (**a**) bentonite and (**b**) laponite (reproduced by permission from ref. [47]). The image width is approximately 1 mm for (**a**) and 500 μm for (**b**).

Laponite, a synthetic clay used in personal care products to modify rheological properties, or as gelator in construction work and artware, also exhibits a similar behaviour. Some exemplary textures of concentrated solutions are shown in Figure 9 for both materials [47]. These are typical nematic textures from disk-like platelets.

Applications of the liquid crystalline phase could again be found in the ordering of inorganic nanosheets by templating LC order for sensors and optical materiuals, or in the application of cosmetics.

#### 2.2.3. Imogolite

**Figure 10:**
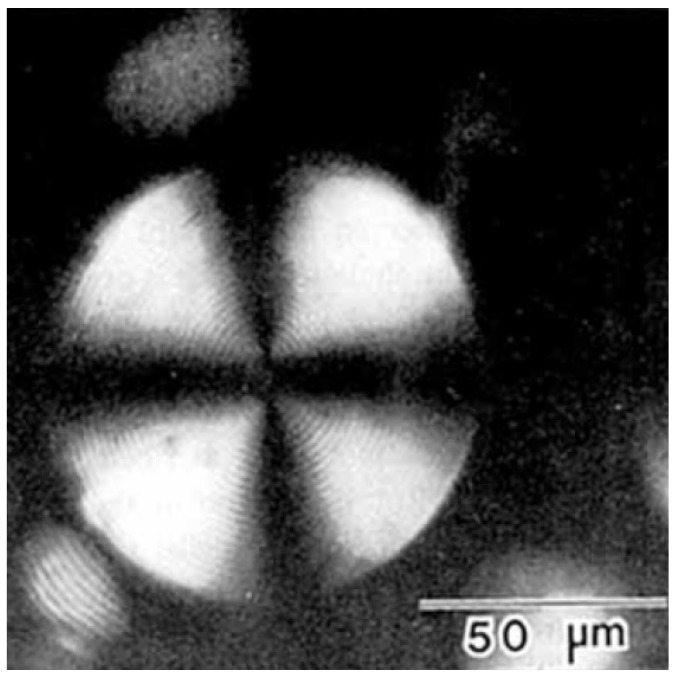
So-called “fingerprint” texture of imogolite, exhibiting an equidistant line pattern, which is somewhat reminiscent of a cholesteric fingerprint texture. Nevertheless, imogolite does not exhibit any chiral constituents, so that the stripe pattern is not indicative of a cholesteric phase. (Reproduced by permission from ref. [49]).

Imogolite is a natural hydrated aluminium silicate found in volcanic ash, which can be dispersed in water under acidic conditions. The observed phase separation between isotropic and anisotropic, birefringent liquid is practically temperature independent [48,49]. Imogolite forms a tubular structure and exhibits a texture reminiscent of a cholesteric fingerprint organisation, demonstrated in Figure 10.

It does not seem to be quite clear why a chiral texture is observed, while the tubes do not seem to exhibit any chirality. Also, the equidistant striped pattern disappears for increasing concentrations of imogolite. Possibly, the texture is related to a banded texture, as it can also be observed for some nematic gels formed from molecular ribbons.

### 2.3. Biological Nanoparticles

Biological or natural fibres, in fact all biological anisotropic nanoparticles, are potential candidates for exhibiting lyotropic liquid crystalline behaviour. For example, cellulose and its derivatives shows cholesteric lyotropic phases in many isotropic solvents [50], and so do cellulose nanocrystals in water [51]. Fibrous or filamentous proteins for example of the collagen family, or muscle proteins like actin can for LC phases. The variety of liquid crystalline behaviour of nucleic acidsDeoxyribonucleic acid (DNA) or ribonuceic acid (RNA) is multiple, and also related rod-like structures, such as the TMV, has long been shown to exhibit mesophases.

#### 2.3.1. Tobacco Mosaic Virus (TMV)

**Figure 11:**
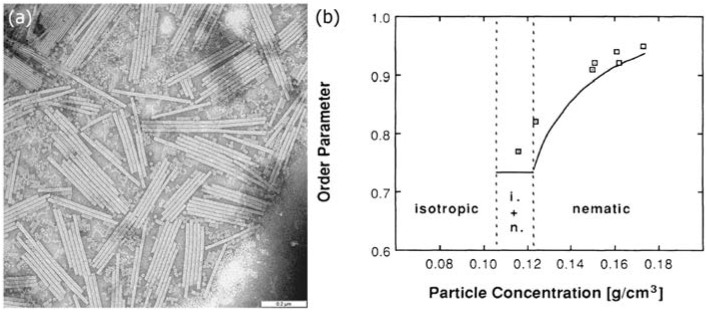
(**a**) Electron microscopic photograph of tobacco mosaic viruses, TMV, indicating an aspect ratio of approximately 15, and a relatively monodisperse length distribution. The scale bar indicates 0.2 μm. Part (**b**) depicts the concentration dependence of the orientational order parameter S, as determined by small angle x-ray scattering, SAXS. The order parameter is zero in the isotropic liquid, and increases from about S ≈ 0.7 to S ≈ 0.95 through the biphasic region and into the regime of the nematic phase at large concentrations. (part (**a**) is reproduced from wikimedia commons, with no author name supplied, while part (**b**) is reproduced by permission from ref. [52]).

The TMV, is a single-stranded RNA virus which affects mainly tobacco plants, but also various other plants, visible by a discolouration of the leaves in a mosaic like pattern. Back in the 1930s it was presumably the first ever virus to be discovered. The TMV can be seen as a natural prototype of a ridged rod. It is very straight and rod-like in structure with a constant diameter of approximately 18 nm and an often uniform length of about 300 nm, thus an aspect ratio of the order of D/L ≈ 15 (see Figure 11a). It is thus ideally suited as a test system for the Onsager theory.

And indeed, in 1936 a first publication [11] reported liquid crystalline behaviour for TMVs at certain concentrations, through the growth of tactoids and with corresponding x-ray investigations (figure with textures). Most likely independently derived, a similar result was reported by Rischkov and Smirnova [53] about five years later. More detailed small angle X-ray scattering (SAXS) experiments were carried out by Oldenbourg and co-workers [52] who produced a small angle diffraction pattern for a magnetic field aligned nematic phase, which allowed the determination of the orientational order distribution function, and thus the scalar order parameter S. The latter indicated a transition from isotropic through a two-phase region into a nematic phase with increasing TMV concentration. The order parameter in the nematic phase changed from about S = 0.75 at the transition to S = 0.95 for high concentrations, which is in accordance with Onsager’s predictions (Figure 11b).

Fraden and co-workers [54] performed a thorough investigation of TMV solutions, measuring the birefringence as a function of concentration, temperature, ionic strength and polydispersity. They observed the appearance of spatial and angular local order for increasing concentration until an isotropic to nematic phase transition is accomplished via an Iso-N two-phase regime. From their measurements, together with a modified Onsager approach, they concluded that the nematic phase stability of TMV suspensions is mainly caused by electrostatic repulsion, rather than attractive (van der Waals) forces between the TMV rods. This indicates a transition due to excluded volume effects as generally predicted by Onsager, whose theory ignores attractive forces all together and is purely based on (steric) repulsion interactions. The complete phase diagram of the tobacco mosaic virus was later predicted from theory and simulations by Graf and Löwen [55], who did not only describe the isotropic to nematic transition with its two-phase region, but also further transitions into smectic phases and colloidal crystal phases.

Tobacco mosaic viruses in the nematic state have also been suggested for the design of silica-TMV mesostructures and nanoparticles, i.e., using the TMV as a template in the synthesis of inorganic frameworks with ordered porosity in order to produce more complicated structures. Fowler et al. [56] describe a method where ordered TMVs in the nematic arrangement are silicated and then thermally removed via biodegradation. This led to silica structures with hexagonally ordered nano-channels of a diameter of approximately 20 nm. The authors also synthesized silica nanoparticles with radially arranged nano-channels. A range of further approaches to use TMV LCs as templates for the controlled synthesis of ordered structures have since been pointed out (see references [57,58] and references therein). It is worth noting, that while the TMV produces nematic ordering, also cholesteric [59] and smectic phases [60] have been observed for different virus suspensions.

#### 2.3.2. DNA

Since the identification of the double helix structure of DNA by Watson and Crick in 1953, based on the X-ray information provided by Gosling and Franklin, this biopolymer has attracted much interest, not only as the carrier of genetic information, but also as a molecule to probe elasticity, as a component in hybrid materials or in bio-nanotechnology. The DNA structure is composed of two helical biopolymers coiled around each other, bound together by hydrogen bonds. Along its length, the structure of DNA is quite flexible and dynamic. The double helix is about 2 nm wide and has a pitch of 3.4 nm, while a DNA molecule can contain millions of base pairs and can have a length in the order of millimetres, and even tens of millimetres. 

**Figure 12:**
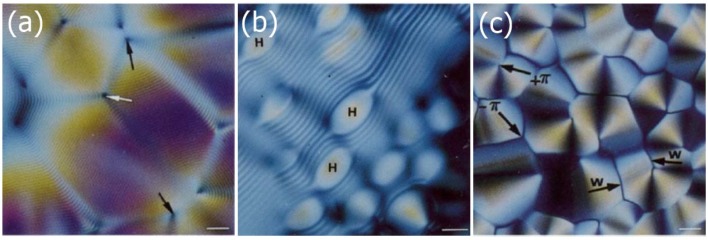
Typical textures observed for the lyotropic phases of relatively long DNA with increasing concentration. (**a**) Cholesteric fingerprint texture with equidistant line pattern due to the helical superstructure of the phase. The distance between two dark lines is equal to identity period of half the pitch, P/2; (**b**) At the transition from the cholesteric to the columnar hexagonal phase; and (**c**) within the fully developed columnar hexagonal phase. The scale bars are 10 μm. (Reproduced by permission from ref. [61]).

The fact that DNA can exhibit liquid crystalline phases has been realized quite some time ago, and there has been an excellent review article of the earlier work until about twenty years ago by Livolant and Leforestier [61]. In 1988, Strzelecka et al. [62] reported on the multiple LC phases of DNA at higher concentrations. Through NMR line width experiments on solutions of DNA fragments of approximately 50 nm in average length, corresponding to 146 base pairs, the isotropic, cholesteric and smectic-like phases, together with their respective two-phase regions iso + chol and chol + smectic were identified as a function of increasing DNA concentration. Furthermore, typical textures were shown, similar to the ones of Figure 12. Already one year later, it was shown by Livolant et al. [63] that the high concentration phase is of the columnar type, and later, by the same authors, it was further demonstrated by electron microscopy of typical double twist cylinders, that the often called “pre-cholesteric” phase was in fact a Blue Phase [64]. The structure and evolution of the liquid crystalline phases of DNA was also confirmed by polarization sensitive two-photon fluorescence microscopy on respectively labelled DNA molecules.

In recent years, the attention of LC forming DNA has slightly shifted towards rather short, more controllable and defined molecules. According to Onsager theory only volume fractions of Ψ > Ψ_Iso-N_ ≈ 4D/L should be able to exhibit a nematic phase. Molecules with aspect ratios L/D < 4 should not show LC phase formation for any concentrations. Nonetheless, Nakata et al. [65] have demonstrated the 6–20 base pair DNA can in fact exhibit cholesteric and columnar phases via end-to-end adhesion and stacking of oligomers into polydisperse, rod-shaped, semi-rigid aggregates, which then act like colloidal particles forming LC phases. This mechanism of self-assembly of short DNA duplexes to form building blocks for cholesteric LCs was detailed later on in subsequent publications [66]. In the original work on short DNA molecules, fully complimentary sequences were employed, which was then extended by Zanchetta et al. [66,67] to partially overlapping sequences and even to LC ordering in systems with a large amount of randomness provided by random DNA sequences, when Bellini et al. [68] discussed liquid crystalline behaviour observed between the isotropic phase of short oligomers and isotropic gels of long random DNA strands. Very recently, this work on very short DNA has been extended to systems with only four base pairs to still show liquid crystalline behaviour via aggregation [69]. An interesting effect for short DNA LCs has been observed for varying concentration, as right handed DNA macromolecules can produce left handed cholesteric structures at low concentration and right handed ones at larger concentrations, passing a structurally non-chiral nematic state as a function of concentration [70]. This is very similar to the temperature induced twist inversion phenomena observed in thermotropic LCs [71,72,73,74,75,76] where in the lyotropic systems the concentrations mimics the role of temperature as the variable of state.

DNA finds its applications in LC research and possible future technology for example in the use as chiral dopants to control the pitch of cholesteric phases [77], as biosensors [78] and even as alignment layers for LC applications [79].

#### 2.3.3. Cellulose Nanocrystals

Cellulose is composed of polysaccharides, linear chains of hundreds to thousands of sugar units. It is a natural polymer which occurs in abundance in nature and has one of the most inexhaustible supplies. Its attractiveness for materials and composites lies in the fact that cellulose is regenerative, easily biodegradable, and optical properties have been studied in great detail. It has long been known that cellulose derivatives in suitable solvents form lyotropic LCs, often with a rather short pitch in the visible range of the spectrum [50,80]. Their phase behaviour and optical properties have been studies in great detail, also with respect to cellulose-based derivatives. Cellulose nanocrystals as hard rod systems seem to have attracted increasing interest only over recent years. 

**Figure 13:**
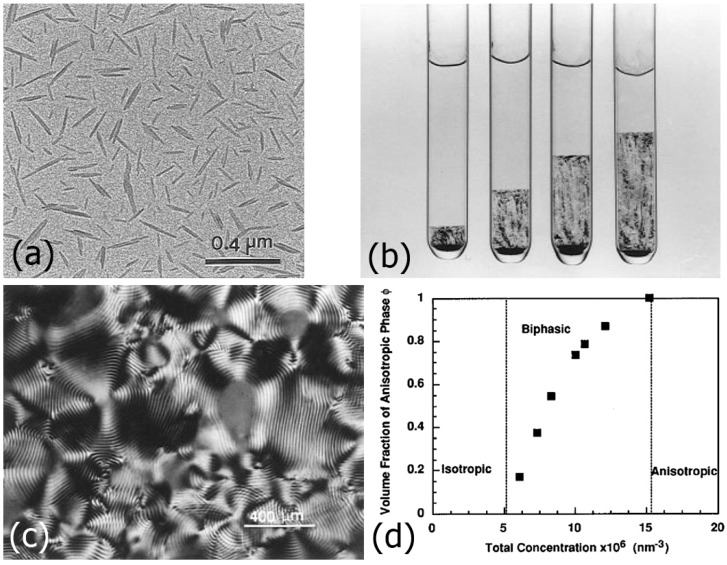
Summary of the basic lyotropic liquid crystalline behaviour of cellulose nanocrystals. (**a**) The nanocrystals of cellulose are composed of chiral polymers and exhibit a length of about 100 nm and lateral dimensions between 10–20 nm, thus aspect ratios in the order of 10; (**b**) For concentration up to about 3% the isotropic phase is observed, which changes to a biphasic region in which the liquid crystalline volume fraction increases with increasing concentration until at about 10–14% a completely anisotropic phase is observed; (**c**) Since the building blocks are chiral, cellulose nanocrystal LCs exhibit a cholesteric phase, as demonstrated by the fingerprint texture. The helical pitch is in the order of 10–20 μm; (**d**) Volume fraction of the anisotropic phase as a concentration of cellulose nanocrystals. (The different parts of the figure were reproduced by permission from ref. [83]).

Like many of the other hard-rod nano-materials we have discussed above, also cellulose nanocrystals form lyotropic LCs in accordance with the predictions of Onsager’s model. Also in this case tactoids may be observed in microscopy of cellulose nanocrystal/water systems. Only here, we are dealing with chiral constituent molecules, such that a chiral nematic or cholesteric phase is observed in contrast to the nematic phases. The cellulose nanocrystals have an average dimension of about 100 nm in length, 25 nm in width and approximately 10 nm in height (Figure 13a). They can thus be seen as lath-like nanoparticles [81]. In the lyotropic cholesteric phase, these nanocrystals orient with their long axis parallel to an average direction, which changes with a continuous twist when proceeding in the direction of the short axis, perpendicular to the long axis. Thus a helical superstructure is formed. For increasing concentration of cellulose nanocrystals, the volume fraction of the anisotropic phase increases, as expected (Figure 13b,d). The observed pitch is generally of the order of 10–20 μm, decreasing with increasing cellulose nanocrystal concentration and increasing with nanocrystal length or aspect ratio [82]. The helical superstructure and pitch is clearly manifested also in the observed textures between crossed polarizers, as an equidistant pattern of dark lines, called a fingerprint texture (Figure 13c).

The general phase behaviour of cellulose nanocrystals in water has been discussed on several occasions [84,85,86,87,88] and results appear to be non-contradictory: below about 3 vol % the solution is completely isotropic. It is followed by a two-phase region of isotropic + LC in the regime of 3–10 vol % nanocrystalline cellulose, and reaches a completely liquid crystalline state at 10–14 vol %. Above 14 vol % a gel is formed [84]. In the two-phase region the anisotropic volume fraction increases with increasing cellulose nanocrystal concentration.

One of the most prominent material parameters of lyotropic cellulose nanocrystal dispersions is the viscosity, which generally increases for increasing concentration and aspect ratio [89]. Also studies relating to the ionic strength have been reported [90], and further the influences of temperature and humidity [91] for dried cellulose nanocrystal films obtained from lyotropic LCs.

Such materials are produced as multifunctional thin films for applications, for example in varying the wavelength of selective reflection across the film diameter, a property which is due to a varying concentration of cellulose nanocrystals [92]. Also the production of plasmonic films of cellulose nanocrystal cholesterics incorporating gold [93,94] or silver [95] nano-rods has been reported. Such composite films display tuneable chiroptic properties.

#### 2.3.4. Active Liquid Crystals

At this point it is worthwhile to also mention active LCs as an emerging topic of pronounced interest. In general, active matter [96,97] resembles a system composed of many active constituents, each of which consumes energy or converts one form of energy to motion or the exertion of a mechanical force. They are therefore intrinsically non-equilibrium systems. Examples are found in a wide variety of soft matter and biological systems, such as swarms of insects, flocks of birds, school of fish, or closer to the topic of this review, bacteria or microtubules. Systems are often of biological origin, but more lately also synthetically derived. They generally show dynamic self-organization and self-propellation. Active LCs have recently become a topic of much increasing interest [98].

In addition, as liquid crystalline systems, active matter is a non-equilibrium system, like cells for example, assemblies of many molecular units working cooperatively to undergo processes like motion, cell division or replication. These systems are actively driven, thus consume energy, which has to be provided from the surrounding. They can thus not be described by equilibrium statistics. The group of Dogic [99] have demonstrated an interesting example of active matter by the use of stretchable microtubule bundles. They showed that active matter can be hierarchically assembled to mimic LCs, but also polymer gels and emulsions by forming an active percolation network at not too small microtubule concentrations. The demonstrated active LCs form the typical s = ±1/2 defects, indicating nematic order with defects that show spatiotemporal dynamics. If one balances the rates of defect creation and defect annihilation, one can achieve steady-state streaming dynamics, which continues over prolonged time scales. This is a behaviour qualitatively different from non-active nematics, as for the latter the defect dynamics follows particular scaling laws for creation [100] and annihilation [101,102] of topological defects. The group of Lavrentovich [103] used a somewhat different active liquid crystal medium. They dispersed motile bacteria, bacillus subtilis, in a liquid crystalline host and demonstrated that the nematic topological defects can be used to command active matter. By employing a variety of different director fields, they showed that the bacteria senses differences in director field deformation. It was observed, that for pure splay and pure bend deformations the bacteria motion is bipolar, with an equal probability distribution for motion along the director field in either direction. This was different for mixed splay-bend regions, where the motion becomes unipolar, directed towards the positive defects and avoiding negative ones. Lavrentovich et al. thus directed the motion of bacteria by the use of defect patterns, and therefore exerting a directing influence on the otherwise chaotic motion. It is very likely that active liquid crystalline systems will become a direction of research where many interesting fundamental aspects are to be discovered, with a high likeliness of future applications in the areas of biotechnology and medicine.

### 2.4. Liquid Crystals from Nanotubes and Nanorods

The largest part of the literature and thus experimental investigations, are related to the dispersion of nanotubes within an already existing LC phase [104,105,106]. This host phase can be nematic, cholesteric or smectic, and already possess a physical functionality, as for example in the form of SmC* ferroelectric liquid crystals (FLC). The aim is to transfer the anisotropic order of the LC onto the dispersed anisotropic particles, the LC acting as a template [27,107]. Due to the properties of the dispersed nanotubes or nanorods, this adds functionality to the dispersion, for example in the form of a switchable conductivity [28]. Orientation of nanotubes and changes of physical properties can also be observed in ferroelectric LCs [108], discotics [109,110], and lyotropic phases [111,112,113,114]. Nevertheless, here we will concentrate on the opposite phenomena, the formation of lyotropic LC phases through the addition of nanotubes and nanorods to an isotropic solvent [115,116,117].

#### 2.4.1. Nanotubes

**Figure 14:**
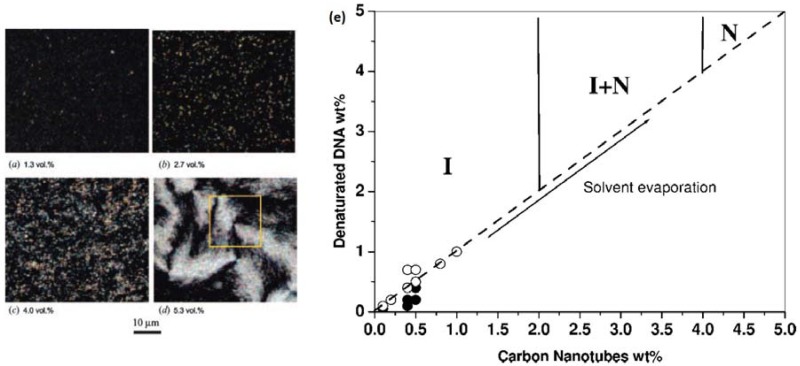
(**a**–**d**) formation of the lyotropic nematic phase of multiwall nanotubes, multiwall nanotubes (MWNT), in water, for increasing concentration through the biphasic region. At approximately 5% by volume, the nematic phase is fully developed as evidenced by a typical Schlieren texture. (Reproduced by permission from ref. [117]); (**e**) Similar results are obtained for DNA functionalized nanotubes. (Reproduced by permission from ref. [121]).

The possible occurrence of liquid crystalline order was first predicted by Somoza et al. [118] who analysed two limiting approaches theoretically: (i) purely attractive van der Waals interactions between the nanotubes, which led to the formation of nematic and columnar phases for increasing concentration of nanotubes; (ii) solely hard-core repulsion, which led to the formation of nematic and smectic A phases for increasing concentration. The isotropic to nematic transition was found to depend on the length of the nanotubes; increasing with increasing nanotube length. Experimentally, it appears that ultra-sonication is of vital importance to de-bundle the nanotubes, increase tube solubility and lead to the observation of lyotropic behaviour. The nematic phase grows in the form of tactoids with an order parameter increasing from about S ~ 0.3 to S ~ 0.5 which increases with increasing time of sonication [119,120], as depicted in Figure 14. It should also be noted that a predicted smectic A phase has not been observed experimentally so far, which can most likely be attributed to the polydispersity of the nanotubes. All in all, the nanotube lyotropic phase formation is quite similar to that of TMVs or DNA.

First experimental evidence for lyotropic nanotube LCs was presented shortly after their prediction, by Song et al. [116,117] for a multiwall nanotube (MWNT) in water system. To enhance the solubility without the need for employing a surfactant, the nanotubes were functionallized with COOH before dispersion in water. The transition from isotropic to the nematic state was observed at a nanotube loading of approximately 1 vol %, with a two-phase region between 1–4 vol %. Above this concentration, a purely nematic state was found [115]. The two-phase region is somewhat wider than that predicted by Onsager, which again can be attributed to the large polydispersity of the MWNTs. Windle and co-workers [122] demonstrated that the longer, straighter nanotubes accumulate in the nematic phase of the dispersion, while impurities, which are always present in nanotube systems, as well as short tubes, accumulate in the isotropic liquid. Badaire followed a similar approach for single-wall nanotubes (SWNT), but instead of covalent functionalization, denatured DNA was adsorbed on the walls of the tubes [115]. The dispersion in water is then facilitated via electrostatic repulsion, as the denatured DNA is charged. This is used to compensate the attractive van der Waals interactions between the nanotubes, and implies that below a certain coating concentration the dispersion remains isotropic. Above the critical coating concentration, a nematic phase is observed above 4 wt % SWNTs, with a two-phase region between 2–4 wt % (see Figure 14e). Electrostatic repulsion to disperse the single-wall nanotubes was also used by Rai et al. [121] for nanotube LCs without the functionalization with chemical groups or decoration with DNA. In this case though, a strong acid had to be chosen as the isotropic solvent, which led to protonation of the tube walls and thus electrostatic repulsion and better tube dispersion.

#### 2.4.2. Nanorods and Nanowires

It appears that it is generally hard to obtain large scale uniformly oriented samples of nanotube based lyotropic LCs. This is probably closely related to the largely unavoidable polydispersity of the systems under investigation. It is likely that a more successful approach may be found in the use of nanorods, which can be produced with a much better monodispersity and where the nanoparticles are straight and less flexible, i.e., behave more like an ideal system in terms of the Onsager description. Systems with dispersed nanorods have been investigated, but again, mainly with respect to dispersions in an already existing (thermotropic) LC. Here the self-organization of the LC is exploited to self-assemble nanorods, to provide added functionality or tuning of physical properties. An example are gold nanorods, LC modified gold particles and gold nanorod LCs [26,123,124,125,126], which enhance the anisotropy of the conductivity, the dielectric constant, and the elastic behaviour.

##### Nanorods of ZnO 

Zinc oxide, ZnO, is generally produced as a white powder for the use in many materials and applications, such as paints, plastics, glass, ceramics, food products and mainly in the rubber industry, where it is employed in the vulcanization process of rubber. It is a wide band-gap semiconductor of the II-VI group and its uses in the electronics industry are in thin-film transistors, light emitting diodes, and as transparent electrodes for liquid crystal displays.

Mostly, ZnO nanoparticles, and other metallic and metal oxide nanoparticles, are incorporated into already existing thermotropic or lyotropic phases, rather than being used to generate the LC behaviour [30]. Lamellar, cubic and hexagonal lyotropic phases have also been reported to be used as a reaction medium in which nanoparticles are synthesised [127]. Reports on the formation of lyotropic LCs from ZnO nanoparticles are comparatively scarce.

**Figure 15:**
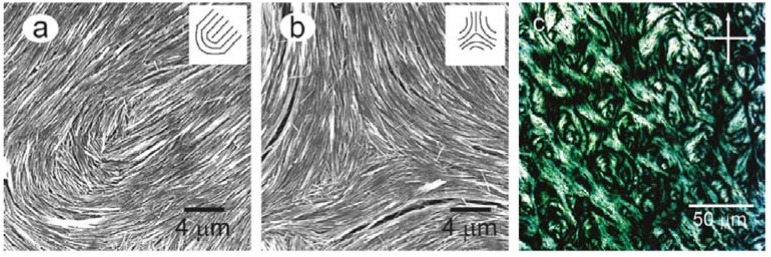
Dried films of ZnO lyotropic nematic phases can be used to image defects of strength (**a**) s = +1/2 and (**b**) s = −1/2; Part (**c**) shows a Schlieren texture of a fully developed lyotropic nematic phase from ZnO nanowires. (Reproduced by permission from ref. [128]).

In the form of single crystal semiconductor nanowires ZnO assembles into lyotropic nematic phases in organic and aqueous solvents. The formation of the LC phase follows that predicted by Onsager, and outlined above, where below certain ZnO nanowire concentrations an isotropic phase is formed, which at higher concentrations becomes a two-phase region and eventually at another, still higher concentration, transforms into a lyotropic nematic phase [128]. For the demonstration of such liquid crystalline behaviour, high aspect ratio nanowires were employed, suitably surface-functionalised by molecules containing sulphur, an alkyl spacer and headgroups such as H or COOH. In the nematic state a nicely developed Schlieren texture can be observed, as shown in Figure 15. On drying thin films from the lyotropic phase, the ZnO nanowires may act like a template of the director field [128], imaging typical s = ±1 and s = ±1/2 defects of the lyotropic nematic phase, similar to polymer stabilized LCs with thermotropic nematic phases [129] (see Figure 15).

The same group of authors also went one step further in the functionalization of ZnO nanowires, by doping with cobalt Co and manganese Mn, to introduce magnetic properties [130]. Also here, high aspect ratio, surface functionalized nanowires were used, and magnetic reorientations of the ZnO director field demonstrated.

##### Nanorods of TiO_2_

Titanium oxide finds its applications in the food industry, as sunscreen, and especially as a white pigment in paper, plastics and paints. In nature it is known as rutile, anastase and brookite, differing in their crystal structure. TiO_2_ nanorods and nanowires are generally produced through a conversion of anastase. Also for TiO_2_ there are reports where lyotropic LC phases are used in the synthesis of nanomaterials, where self-assembled lamellar, spherical and rod-like structures may be observed [131]. Reports of TiO_2_ nanowires being used to generate lyotropic phases are scarce [132]. One such report describes a two-stage assembly process in the formation of a lyotropic nematic phase, by first forming a primary structure, such as ribbons, which then in a second self-assembly step through an increase in concentration may form a nematic and lamellar lyotropic LC.

##### CdSe Semiconductor Nanorods

Cadmium selenide nanorods are semiconductors, typically of length ~40 nm and width ~6 nm. This means they have an aspect ratio for which one can well expect the formation of orientational order as it is observed for nematic phases [133]. Due to the fact that these nanoparticles can also be produced with an excellent monodispersity, one even has the opportunity to possibly detect smectic ordering, i.e., the formation of at least one dimensional positional order [134].

**Figure 16:**
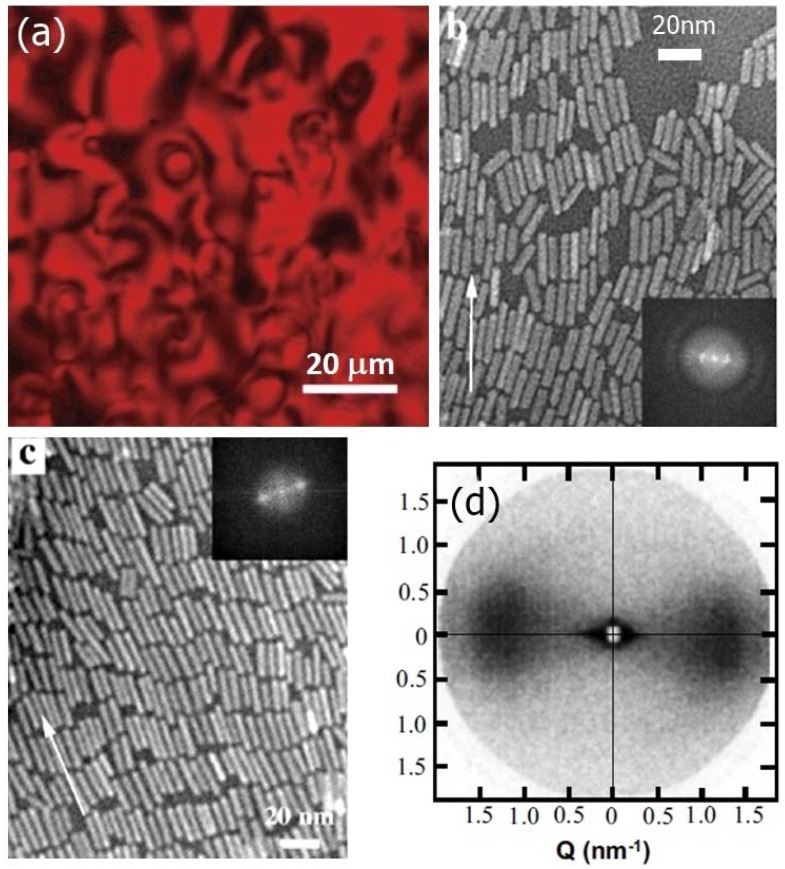
CdSe nanorods exhibit typical nematic Schlieren textures, as shown in part (**a**) of the figure (reproduced by permission from ref. [133]). The electron micrographs of parts (**b**) and (**c**) indicate nematic and smectic ordering, thus orientational and additional one-dimensional positional order, respectively. The insets show the corresponding Fourier transforms. (Reproduced by permission from ref. [135]); (**d**) depicts the SAXS picture of the nematic phase, which also clearly evidences orientational order. (Reproduced by permission from ref. [134]).

CdSe nanorods show indeed a pronounced appearance of lyotropic nematic phases in the presence of organic solvents [134], as shown in the distinct Schlieren textures of Figure 16a, where s = 1/2 and s = 1 disclinations are observable. Also small angle X-ray experiments on oriented samples nicely present evidence for nematic ordering (Figure 16d), while transmission electron microscopy (TEM) reveals not only a nematic structure of nanorods, but also positional order for higher concentrations (Figure 16b,c). Liquid crystalline self-assembly of nanorods has been reviewed recently by Thorkelsson et al. [136]. Not only have semiconducting nanorods been investigated for the formation of lyotropic LCs, but also within LC templates [31].

### 2.5. Liquid Crystals from Nanoplates

Just like the disk-like colloidal structures of for example clay particles, also other materials of that shape can exhibit very stable lyotropic LC phases as a function of particle concentration. One of the most prominent examples are the derivatives of graphene [137,138,139,140,141]. Graphene has attracted much attention over the recent years due to its promising properties in terms of elastic modulus and conductivity while only exhibiting flakes of the nanometer to micrometer size which are only a carbon monomolecular thick.

#### 2.5.1. Graphene

In terms of liquid crystalline behaviour graphene itself is actually not the material of choice, due to its poor solubility and dispensability in isotropic solvents. This has been tested for a large variety of solvents with varying polarity [142] and it appears that solubility is slightly increased for increasing dielectric constant. Nevertheless, overall the solubility of graphene in any solvent is very small and concentrations to observe lyotropic liquid crystalline behaviour are not easily achieved, not even with prolonged ultrasonication to avoid aggregation and coagulation.

A possible way forward are protonated graphenes. The formation of a lyotropic liquid crystalline phase formed by graphene in chlorosulphonic acid was first reported by Pasquali and co-workers [143] in 2010. A nematic texture was observed to indicate liquid crystallinity. The structure of the nematic phase is similar to that of a discotic nematic phase, with the director being normal to the plane of the graphene sheets. Despite the principle demonstration of liquid crystalline behaviour, processing of these systems is obviously not desirable, and systems with more environmentally friendly solvents and better solubility need to be found.

#### 2.5.2. Graphene Oxide

**Figure 17:**
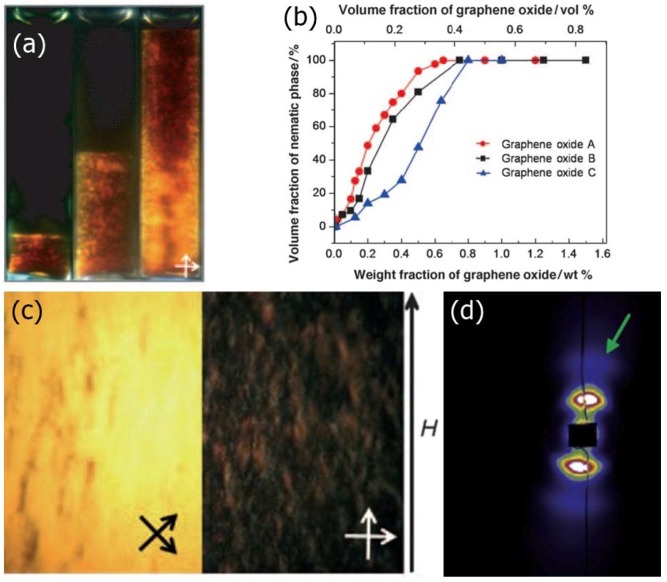
(**a**) Qualitative illustration of the increasing LC volume fraction for increasing graphene oxide, GO, concentration; Part (**b**) quantifies this behaviour for three graphene oxide samples from different sources. The difference in quantitative behaviour as the biphasic concentration regime is passed, is due to a variation of polydispersity and graphene flake size among other influences; (**c**) Application of magnetic fields can be used to uniformly orient the lyotropic nematic phase of graphene oxide, which is evidenced by rotation of the sample between crossed polarizers; (**d**) Also with small angle X-ray diffraction one can demonstrate orientational order of the director, obtained in capillaries. (Parts (**a**–**c**) are reproduced by permission from ref. [144], while part (**d**) is reproduced by permission from ref. [145]).

This is the case with graphene oxide (GO), which represents a form of graphene decorated with hydroxyl, carboxyl and epoxide groups. This makes it easily dissolvable in water and other solvents. A further advantage of graphene oxide is the fact that in contrast to graphene, it is readily available in large quantities at a very reasonable price. As first demonstrated by Kim and co-workers [144] and Xu and Gao [145] in 2011, GO in water or organic solvents forms a nematic phase above a certain threshold concentration, with typical textures observed, as shown in Figure 17a for increasing GO concentration. As common, a two-phase behaviour is observed as demonstrated also in Figure 17b for three different graphene oxide sources. The formed phase is very stable with respect to temperature, up until the boiling point of the solvent.

Xu and Gao [145] actually claim that the phase they observe can be described by the model of the twist grain boundary (TGB) phase, where blocks of smectic layers are rotated with respect to each other, while the grain boundaries between blocks are arrays of screw dislocations. The rotation of the blocks will eventually lead to a helical superstructure, which can be commensurate or incommensurate. They attribute their interpretation to the observed weak layering by small angle X-ray scattering (Figure 17d) in combination with LC texture observation and cryo-Scanning Electron Microscopy. This appears to be a point of controversy, because the formation of a TGB-like phase requires the presence of chirality, which is absent in the studied system, as neither the graphene oxide, nor the solvent are chiral. The observed textures also appear different than the common fingerprint textures observed for chiral nematic or cholesteric LCs, without a clear periodicity, appearing more like textures observed in shear banding. In addition, the fact that the graphene oxide sheets exhibit a large polydispersity makes it less likely to form a TGB structure. At this point the detailed structure of the observed phase does not seem to be quite clear, and possibly further investigations will be needed. Nevertheless, it is without doubt that the observed aqueous graphene oxide suspensions exhibit liquid crystalline behaviour.

It should further be pointed out, that the actual phase appearance or in fact possibly the diagram slightly depends on the average size of the GO flakes, the polydispersity, the dielectric constant of the solvent and confinement conditions [146]. The liquid crystalline phase is formed at lower concentrations for larger GO flake sizes, it is observed more easily for solvents with an increased dielectric constant, such as water, and it is somewhat suppressed or not observable for more confined geometries. This is most likely due to the very strong planar anchoring of the graphene oxide sheets to the bounding glass substrates, which produces pseudo-isotropic behaviour. The fact that liquid crystalline behaviour of GO can be observed at much lower concentrations if the flakes exhibit a larger size was also observed by Dan et al. [147]. Furthermore, the LC formation in dependence of different organic solvents has been discussed by Jalili et al. [148]. At last, an interesting scenario can be observed if dispersing graphene oxide in a thermotropic nematic. Increasing the temperature above the clearing point, converts the host LC into an isotropic phase, which can then in combination with the GO act as a solvent to form a lyotropic nematic phase [29]. One can thus observe the transition between a thermotropic and a lyotropic nematic phase, which can be shown by dielectric spectroscopy, but is not observable in differential scanning calorimetry, thus apparently not connected to a latent heat [149].

**Figure 18:**
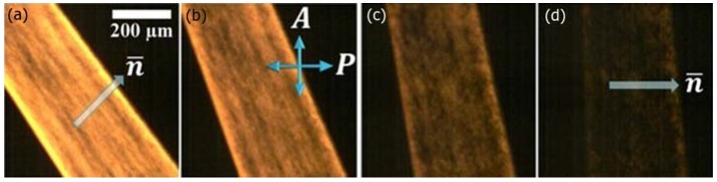
Confinement in channels of plain, untreated glass, can also provide a simple mechanism of orientation for the lyotropic nematic phase of graphene oxide. The transmission of the LC changes with a periodicity of 90° when rotated between crossed polarizes. It is brightest when the director is oriented at 45° to either of the polarizers (**a**) and darkest, when it is parallel to either polarizer A or P (**d**); In between, the transmission continuously varies (**b**,**c**). (Reproduced by permission from ref. [146]).

Graphene oxide LCs can be oriented by magnetic field application, as shown in Figure 17c with the corresponding small angle X-ray scattering image showing the typical pattern of orientational order (Figure 17d). Under the confinement of LC sandwich cells, graphene oxide nematic can be oriented between untreated glass plates or in channels, such that the GO plane lies parallel to the substrates [146]. The director therefore is oriented normal to the substrate plane, and the sample can be rotated between well oriented bright and dark states between crossed polarizers (see Figure 18).

Song et al. [150] have demonstrated that application of an AC electric field to a lyotropic graphene oxide nematic LC can result in electro-optic switching, based on the Kerr effect, with a very large Kerr coefficient. This effect can also be used to orient graphene oxide sheets [151].

#### 2.5.3. Reduced Graphene Oxide

Heating graphene oxide above approximately 165 °C thermally reduced GO to rGO, which results in a partial recovery of graphene properties, especially the electronic ones, but at the cost of solubility, which in turn increases the tendency for aggregation and coagulation, making it more difficult to obtain liquid crystalline behaviour. This can be compensated by employing surfactants to stabilize the rGO flakes, as demonstrated by Poulin et al. [152]. One may thus partially maintain the favourable electronic properties of graphene, while additionally being able to exploit the self-organization due to liquid crystallinity.

#### 2.5.4. Other 2D Materials

One could expect that other two-dimensional materials similar to graphene, graphene oxide or reduced graphene oxide, such as boron nitride, indium selenide or gallium selenide, MoS_2_, NbSe_2_, WO_3_ or WS_3_ can also exhibit lyotropic LC phases at certain concentrations in suitable solvents, especially if these materials occur in single layers. This will then most likely have strong parallels to inorganic LCs and clays.

## 3. Summary and Outlook

The topic of LCs and nanomaterials has attracted increasing attention over the last years, not only within the LC community, but also more broadly as soft materials in general and model anisotropic colloid systems. An extensive summary of up-to-date knowledge can be found in the two-volume book by Lagerwall and Scalia [153,154,155]. The three main reasons for this increased interest are (i) nanomaterials in thermotropic LCs can be used to add functionality and tune the properties of the liquid crystalline system; (ii) Phases, especially those of the frustrated type, can be stabilized, and novel materials with anisotropic properties can be created, which spontaneously align shape anisotropic nanoparticles. This can be achieved either through templating liquid crystalline order from thermotropic, as well as lyotropic phases, as well as the formation of lyotropic phases themselves, by nanoparticles ordering in an isotropic solvent; (iii) LC—nanoparticle composites, may these be of the thermotropic or the lyotropic type, allow for the construction of nanotechnology devices in many diverse areas, such as displays, sensors, biological engineering, or even functional clothing. In this review, we have tried to give a broad overview of different lyotropic liquid crystalline systems, based on a variety of anisotropic particles in the colloidal size range. These can be one- or two-dimensional nanomaterials. In both cases, initial investigations on inorganic materials go back for about a century, although they have by far not attracted the attention of their organic, thermotropic counterparts, which is mainly due to the success of the latter in electro-optic and display devices. One of the classic examples of inorganic LCs [20] is vanadium pentoxide, V_2_O_5_, which dates back to about 1915. While many inorganic LCs are formed by one-dimensional nanoparticles, mineral and clay LCs [44,46] are mostly obtained from plate like, thus two-dimensional particles. The classic examples of biological lyotropic LCs are the TMVs [11] (and other similar viruses), as well as DNA [61]. One material which is located at the borderline between biological one- to two-dimensional crystals, are cellulose nanocrystals [86]. An increasing amount of literature on recent further lyotropic LCs can be found for carbon nanotubes [106,116] (as well as similar nanotubes and nanowires), and graphene oxide [154].

All of the above discussed lyotropic liquid crystalline systems from rods or plates have one feature in common: they all obey the theoretical description initially formulated by Onsager in the 1940s, at least to a large extent, and often even quantitatively. This has also been demonstrated by a variety of computer simulations and experimental work summarized in references [18,19,155].

Given the synthesis and development of ever new nanomaterials, and the rapid advancement of nanotechnology, it seems to be out of question that lyotropic, anisotropic particle based LCs will play an increasing role of importance in the future. This is mainly due to the fact that many of the functionalities observed and exploited in thermotropic LCs, like electric and magnetic reorientation, and with it a change of birefringence, ferroelectricity or magnetic properties, can now also be observed in lyotropic LCs. The properties of self-assembly, self-organisation, and spontaneous alignment will be beneficial for nanotechnological applications, and the fact that for many of the lyotropic systems, water can be used as a solvent, favours environmentally friendly production mechanisms, which are clearly the way forward for future applications.
