# Correction: Dierking, Ingo and Al-Zangana, Shakhawan. Lyotropic Liquid Crystal Phases from Anisotropic Nanomaterials. *Nanomaterials* 2017, *7*, 305

**DOI:** 10.3390/nano8010045

**Published:** 2018-01-15

**Authors:** Ingo Dierking, Shakhawan Al-Zangana

**Affiliations:** 1 School of Physics and Astronomy, University of Manchester, Oxford Road, Manchester M13 9PL, UK; 2 College of Education, University of Garmian, Kalar 46021, Iraq; shakhawan.al-zangana@hotmail.com

Due to an oversight during production, the authors wish to make the following correction to reference [65] of this paper [1]. The published reference [65] was incorrect. The correct reference [65] is shown below.

65. Nakata, M.; Zanchetta, G.; Chapman, B.D.; Jones, C.D.; Cross, J.O.; Pindak, R.; Bellini, T.; Clark, N.A. End-to-End Stacking and Liquid Crystal Condensation of 6–to 20–Base Pair DNA Duplexes. *Science* **2007**, *318*, 1276–1279.

The authors regret any inconvenience or misunderstanding caused by this error. The manuscript will be updated and the original will remain available on the article webpage.
